# A toxin antitoxin system promotes the maintenance of the IncA/C-mobilizable *Salmonella* Genomic Island 1

**DOI:** 10.1038/srep32285

**Published:** 2016-08-31

**Authors:** Kevin T. Huguet, Mathieu Gonnet, Benoît Doublet, Axel Cloeckaert

**Affiliations:** 1 INRA, UMR1282, Infectiologie et Santé Publique, F-37380 Nouzilly, France; 2 Université François Rabelais de Tours, UMR1282 Infectiologie et Santé Publique, F-37000 Tours, France

## Abstract

The multidrug resistance *Salmonella* Genomic Island 1 (SGI1) is an integrative mobilizable element identified in several enterobacterial pathogens. This chromosomal island requires a conjugative IncA/C plasmid to be excised as a circular extrachromosomal form and conjugally mobilized *in trans*. Preliminary observations suggest stable maintenance of SGI1 in the host chromosome but paradoxically also incompatibility between SGI1 and IncA/C plasmids. Here, using a *Salmonella enterica* serovar Agona clonal bacterial population as model, we demonstrate that a Toxin-Antitoxin (TA) system encoded by SGI1 plays a critical role in its stable host maintenance when an IncA/C plasmid is concomitantly present. This system, designated *sgiAT* for *Salmonella* genomic island 1 Antitoxin and Toxin respectively, thus seems to play a stabilizing role in a situation where SGI1 is susceptible to be lost through plasmid IncA/C-mediated excision. Moreover and for the first time, the incompatibility between SGI1 and IncA/C plasmids was experimentally confirmed.

The *Salmonella* genomic island 1 (SGI1) is a chromosomally-located island that may carry several antibiotic resistance genes and was firstly identified end of the 1990s in the multidrug-resistant epidemic clone of *Salmonella enterica* serovar Typhimurium (*S*. Typhimurium) DT104^1^,^2^,^3^. This island is 43 kb in size and is found integrated most of the time within the last 18 bp of the chromosomal *trmE* gene (also named *thdF*). SGI1 has been demonstrated to be an integrative mobilizable element (IME), lacking some functional genes to be self-transferable and which are supplied by conjugative plasmids of the IncA/C family^4^,^5^,^6^,^7^,^8^,^9^,^10^. Only this plasmid family has been shown to be able to mobilize SGI1 into several host strains^9^.

SGI1 classically contains a complex class 1 integron, named In104 according to its initial host strain^11^, located at the 3′ end of the island. However, since its first identification SGI1 has been reported to carry many other different class 1 integrons, including other or additional resistance genes increasing its MDR phenotype and to be found in many other epidemic *S. enterica* serovars. These antibiotic resistance gene cluster variants have been classified from SGI1-A to the latest one SGI1-Z^12^, in the order of their discovery. On the other hand, since 2006 SGI1 and related islands have also been identified in *Proteus mirabilis* clinical and environmental isolates. The number of reported cases of SGI1 variants and closely related islands such as PGI1 (for *Proteus* genomic island 1) is also increasing in this bacterial species^12^,^13^,^14^,^15^,^16^,^17^,^18^,^19^. Of particular concern for public health is the emergence of *P. mirabilis* strains carrying SGI1 or related islands with extended-spectrum β-lactamase and/or metallo-β-lactamase genes^12^,^15^,^16^,^17^,^18^,^19^. Thus understanding molecular mechanisms by which SGI1 spreads in bacterial populations may help implementing measures or strategies to combat further dissemination of this island. It implicates also understanding its intimate relationship with other mobile genetic elements such as plasmids of the IncA/C family required for mobilization of this island^4^,^5^,^6^,^7^,^8^,^9^,^10^.

While several essential functional genes or regulatory genes have been experimentally uncovered in this relationship promoting the transfer of SGI1^4^,^5^,^8^,^10^, some observations raise other questions. Among these is the fact that to our knowledge SGI1 and IncA/C plasmids have not been found together in clinical isolates. It thus raises the question if SGI1 and IncA/C plasmids are able to maintain together along bacterial generations, although their functional complementarity seems essential for the transfer of SGI1. Among other unanswered observations is also the high stability of SGI1 in the chromosome once acquired. It was suggested in the first report on SGI1 in 2000 where the authors were unable to detect the loss of SGI1 by PCR in a Canadian *S*. Typhimurium DT104 isolate^1^. More recently Kiss *et al*. studied more deeply the stability of SGI1 but they were also unable to detect any cell that had lost SGI1 after 350 generations and more than 16,000 clones tested^7^. This high stability suggested that SGI1 may encode a mechanism that is able to stabilize it within the host cell. Among stabilization systems the most classical known to date for genomic islands are the toxin-antitoxin (TA) systems^20^,^21^,^22^,^23^,^24^. They were originally discovered on low-copy-number plasmids, and have been more recently described on integrative conjugative elements^25^,^26^. TA systems contribute to the maintenance of genetic elements by killing or growth arrest of daughter cells that do not inherit a copy of the genetic element during bacterial division. This phenomenon is called Post Segregational Killing (PSK)^27^,^28^. The mechanism underlying PSK is based on a different stability of the toxin and its cognate labile antitoxin proteins usually encoded by class I or II TA operons^28^.

To investigate the possible mechanisms involved in the stable chromosomal maintenance of SGI1, we firstly assessed the role of a putative TA system encoded by SGI1. As an interplay with plasmids of the IncA/C family is also suspected in the SGI1 maintenance, we also studied stability of SGI1 and its putative TA system in presence or not of an IncA/C plasmid.

## Results and Discussion

### The SGI1 S026-S025 open reading frames encode a functional TA system

**Figure 1:**
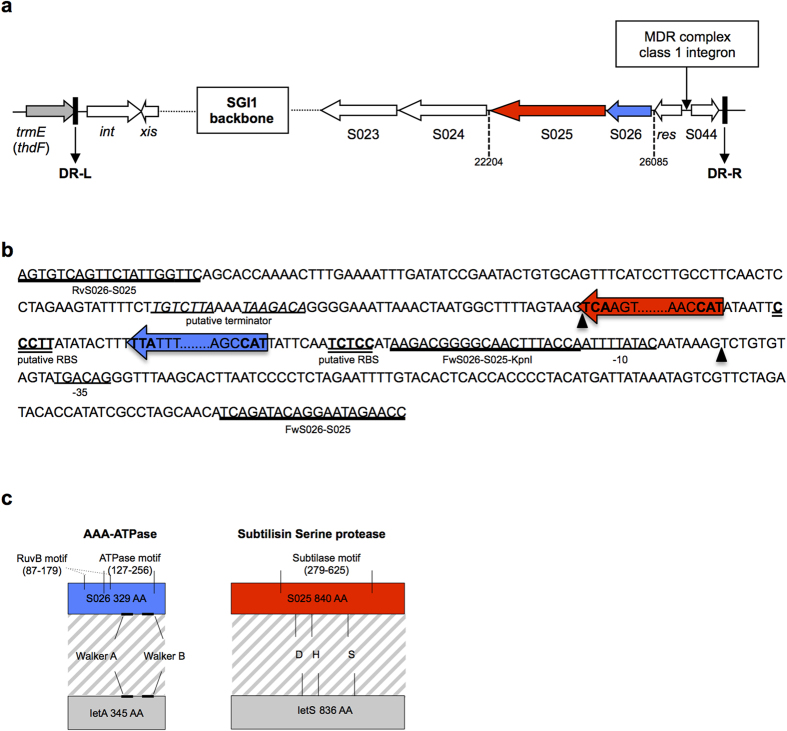
Schematic representation of the SGI1 S026-S025 region and amino acid sequence analysis of the deduced proteins. (**a**) Schematic view of the genetic environment of the S026 and S025 open reading frames in the SGI1 backbone. ORFs S026 and S025 are highlighted in blue and red, respectively. DR-L and DR-R are the 18-bp left and right direct repeats, respectively, bracketing SGI1. The grey arrow represents the chromosomal gene *trmE* (also called *thdF*) in which SGI1 integrates in a site-specific manner at the 3′-end. The crucial genes *int* and *xis* for excision/integration of SGI1 in the chromosome are indicated. (**b**) Nucleotide regions of interest are detailed. S026 and S025 are separated by 20 bp and would thus constitute an operon and transcribed in this order. The putative promoter region (-35 -10 boxes), putative ribosome binding sites (RBS) for each orf, and putative transcriptional terminator region are indicated. Primer sequences (RvS026-S025, FwS026S025-KpnI, FwS026-S025) used for plasmid constructions are underlined. Black triangles delimit the total deletion in 959SA97ΔS026S025 (**c**) Amino acid sequence analyses of S026 and S025. The homologies and conserved motifs with the corresponding IetS (toxin) and IetA (antitoxin) encoded by the pTIC58 plasmid from *Agrobacterium tumefaciens* are indicated. The deduced amino acid sequence of S026 shows 50% identity (63% similarity) with the antitoxin IetA and the product of S025 harbouring a weaker identity of 36% (51% similarity) with the corresponding toxin IetS.

**Figure 2:**
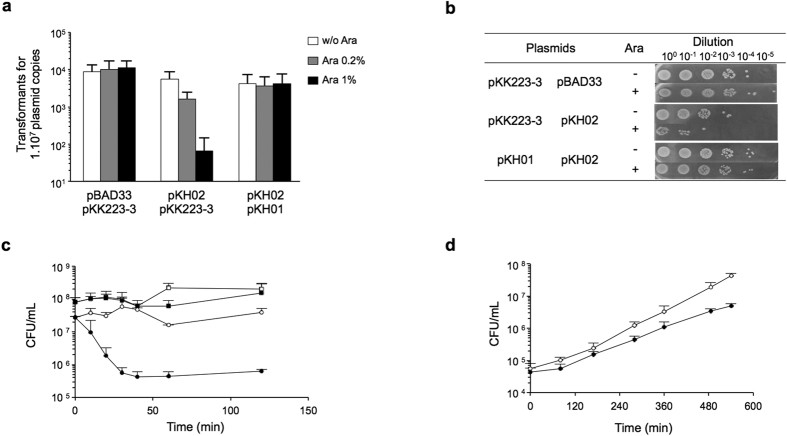
The SGI1 S026-S025 open reading frames encode a functional TA system. (**a**) Transformation efficiency of the pBAD33 plasmid vectors or its pKH02 derivative expressing the putative toxin S025 under the control of the arabinose-inducible *P*_*BAD*_ promoter in *E. coli* DJ480 strains carrying the pKK223-3 or its derivative expressing the putative antitoxin S026 (plasmid pKH01). The efficiency of transformation is represented by the number of transformants obtained per 10^7^ plasmid copies of pBAD33 or pKH02 for *E. coli* strains expressing or not the putative antitoxin S026. Expression of S025 was induced or not by arabinose concentrations of 0%, 0.2%, or 1%. (**b**) Toxicity-antitoxicity assays on LB agar. Drops of 10-fold serial culture dilutions of *E. coli* strains carrying the plasmid combinations obtained in (**a**) were deposited on LB agar medium with or without arabinose at a final concentration of 1%. After overnight incubation at 37 °C plates were photographed. Presence/absence of arabinose (Ara) is represented by +/− symbols. (**c**) Kinetics of toxic action of S025 (pKH02) and its counteraction by S026 (pKH01) in *E. coli* DJ480. Empty squares: *E. coli* carrying plasmid combination pKH02-pKH01 cultured in the absence of arabinose; filled squares: the same cultured in the presence of arabinose at 1%; empty circles: *E. coli* carrying plasmid combination pKH02-pKK223-3 cultured in the absence of arabinose; filled circles: the same cultured in the presence of arabinose at 1%. S025 shows toxic activity rapidly in less than 30 min. on the *E. coli* host strain grown in the presence of arabinose whereas this toxic action is fully counteracted when S026 is present. (**d**) PSK assays *in E. coli* TOP10 cultured in RM medium at 42 °C supplemented with 2.5% arabinose. Empty circles: growth curve of *E. coli* carrying pMLO59 empty vector; Filled circles: growth curve of *E. coli* carrying pKH04 (pMLO59 containing S026-S025 under the control of the *P*_*BAD*_ promoter). A PSK effect is observed after 180 min of growth. In panels (a,c,d), values correspond to the means of results for three independent experiments. Error bars represent standard deviations.

Preliminary observations have suggested that SGI1 may encode a stabilization system playing an important role in its stable chromosomal maintenance^7^. Interestingly, among the uncharacterized SGI1 orfs there are two, i.e. S025 and S026, that may encode a TA stabilization system according to similarity with the deduced amino acid sequences of a TA system identified on the tumor-inducing plasmid pTiC58 from *Agrobacterium tumefaciens*^29^. The SGI1 orfs S025 and S026 are respectively schematized with their characteristics in Fig. 1. S025 shows similarity to subtilisin serine protease and would encode the toxin while S026 would encode the antitoxin showing similarity to the AAA-ATPase family of proteins. The role of SGI1 S026-S025 as a TA system was firstly assessed in *E. coli* using plasmid vectors and methods conventionally used for functional characterization of TA systems as described in the Materials and Methods section. First, the transformation efficiency of plasmid vectors expressing the putative toxin S025 (plasmid pKH02) was assessed into *E. coli* strains carrying either the empty vector pKK223-3 or its pKH01 derivative expressing the putative antitoxin S026. As shown in Fig. 2a transformation efficiency of plasmid pKH02 expressing S025 was reduced, relative to the empty plasmid vector pBAD33, by 100- to 1000-fold when expression was induced with arabinose at concentrations of 0.2% or 1%, respectively. On the other hand, under the same conditions these reductions were not observed when plasmid pKH01 expressing the putative antitoxin S026 was present, thus suggesting that S026 counteracts the toxic activity of S025. Serial dilutions of each *E. coli* strain of this experiment spotted on LB plates in the presence or absence of arabinose showed also these effects to the same extent as the transformation efficiency test (Fig. 2b). Figure 2c shows the kinetics of toxic action of S025 (pKH02) and its counteraction by S026 (pKH01) in the *E. coli* host strains. The induction of S025 transcription shows toxic activity rapidly in less than 30 min on the *E. coli* host strain in the absence of S026 whereas viability is not affected when S026 is present (Fig. 2c). Finally, the entire putative operon S026-S025 was unable to mediate a PSK effect when cloned in a replication-thermosensitive plasmid and expressed from its own putative promoter (Supplementary Fig. 1). However, when expression of the S026-S025 orfs was induced in plasmid pKH04, a slight defective growth of the *E. coli* host strain could be observed in this PSK assay (Fig. 2d).

All together above results clearly show that the SGI1 S025-S026 orfs encode a functional TA system, where the S025 subtilisin serine protease homologous protein encodes the toxin and the S026 AAA-ATPase homologous protein the antitoxin. The results obtained are qualitatively and quantitatively in agreement with those previously published for other TA systems^29^,^30^,^31^,^32^, of which the closest is the TA system of plasmid pTiC58 from *Agrobacterium tumefaciens*^29^. Therefore S026 and S025 characterized in this study were designated *sgiA* and *sgiT* (for *Salmonella* genomic island Antitoxin and Toxin, respectively). The fact that *sgiAT* does not induce a PSK effect when expression is under the control of its own putative promoter suggests that it requires positive activation to be expressed. These results also suggested that a sufficient expression level of *sgiAT* is necessary to exert its role in stabilization of SGI1 or other mobile elements carrying it. Moreover, the apparent PSK effect is also probably dependent on the quantity as well as on the stability of both proteins. Thus possibly, expression of this TA system is finely tuned to exert its role in stabilization. The mechanisms of regulation of expression and possible inducers or inducing conditions remain to be identified.

### Incompatibility between SGI1 and IncA/C plasmids and requirement of the *sgiAT* system for stable maintenance of SGI1 in the presence of an IncA/C plasmid

Some preliminary observations have previously suggested incompatibility between SGI1 and IncA/C plasmids. Among them at the epidemiological level, is the fact that both elements have not been reported together in epidemic MDR *Salmonella* clinical or environmental isolates^33^. Recent examples are provided with the global spread of the MDR *S. enterica* serovar Kentucky ST198 clone^34^,^35^. This clone, which in addition is highly resistant to ciprofloxacin, harbors most of the time the variant SGI1-K or derivatives of it responsible for the MDR phenotype. Interestingly, for two recent carbapenemase-producing isolates of this epidemic clone, with the same ciprofloxacin-resistant ST198 background, the carbapenem resistance genes were found to be carried on the one hand by an IncL/M plasmid in a strain carrying SGI1-K and on the other hand by an IncA/C plasmid in a strain lacking SGI1^36^. Presumably in the latter strain SGI1 may have excised due to a possible incompatiblity with IncA/C plasmid, although we cannot provide evidence for this because currently molecular signatures for excision have not been identified or do not exist. Moreover, experimentally during conjugation experiments both elements have also not been found to occur together in transconjugant strains^6^,^4^,^8^,^10^.

**Figure 3:**
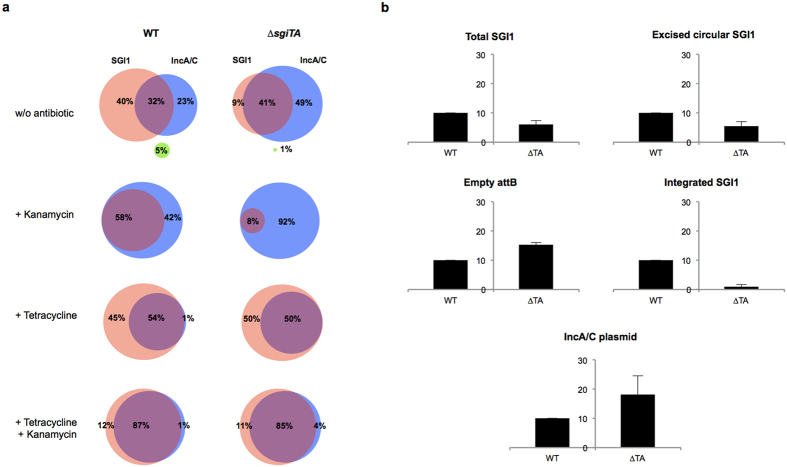
Impact of *sgiAT* on SGI1 stability and incompatibility with IncA/C plasmid in *S*. Agona. (**a**) Results are represented as proportions (%) of *S*. Agona 959SA97 bacterial cells harbouring SGI1, the IncA/C plasmid R55 or both elements following culture as described in the Materials and methods section of an initial population constituted of 10 pooled colonies, and plated after culture on LB plates with or without antibiotics. Tetracycline and kanamycin are selective for SGI1 and the IncA/C plasmid R55, respectively. The colours indicate the following populations 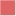 SGI1 only, 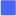 IncA/C only, 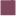 both SGI1 and IncA/C, 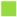 neither SGI1 nor IncA/C. These results were obtained from 3 independently repeated experiments. (**b**) qPCR quantification of SGI1 and IncA/C plasmid R55 on total DNA of 10 pooled colonies isolated on LB plates without antibiotics. The results were normalized to the copy number of chromosome for each sample and expressed as arbitrary units (a.u.), the wild-type strain being placed at 10 a.u. Results were obtained from 3 independently repeated experiments. All differences between the SGI1^WT^ and SGI1^Δ*sgiAT*^ strains are statistically significant (Student T-test: *p* < 0.001).

To assess the role of TA in stable maintenance of SGI1 in the chromosome, *sgiAT* was deleted from SGI1-carrying *S*. Agona strain 959SA97 (Table 1) and stability tests were performed according to Kiss *et al*.^7^ (Table 2). To our surprise, despite the lack of *sgiAT*, no loss of SGI1 could be detected after 350 bacterial generations for the mutant SGI1^Δ*sgiAT*^ strains as for the control strain where the crucial *int* gene for excision/integration had been deleted resulting in a SGI1 island unable to excise from the chromosome and thus that cannot be lost (Table 2)^6^. However, these assays were carried out in strains lacking an IncA/C plasmid representing a major bias, since the excision of SGI1 is triggered by the transcriptional regulator AcaCD encoded by IncA/C plasmids^5^,^8^. Thus, in absence of an IncA/C plasmid the excision of SGI1 is below the detection limit of qPCR (<10^−6^/cell), and suggests that under this condition the stable maintenance of SGI1 is fully ensured by its chromosomal integration^6^. Therefore, we developed a SGI1 stability experiment in the presence of the IncA/C plasmid R55, used previously as helper plasmid to mobilize SGI1^6^. Unexpectedly, heterogeneous bacterial populations were obtained consisting of cells containing either both SGI1 and IncA/C plasmid R55, the one or the other element, or none of them and that thus had lost both elements (Fig. 3). More precisely, without antibiotic selective pressure, only 32% of the bacterial population was shown to carry both elements in the R55- and SGI1^WT^-carrying strain. Fourty percent and 23% of the bacterial population carried SGI1^WT^ alone and R55 alone, respectively. These data indicate that SGI1 and the IncA/C plasmid R55 cannot maintain together in the *S*. Agona bacterial population tested confirming the incompatibility between these genetic elements. In addition, deletion of *sgiAT* resulted in a shift towards loss of SGI1 from the bacterial population in presence of IncA/C plasmid R55 and without antibiotic selective pressure. More precisely, under these conditions the bacterial population carrying SGI1 decreased from 40% to 9% (Fig. 3a). Concomitantly the percentage of cells carrying only the IncA/C plasmid R55 increased from 23% to 49%. The bacterial population carrying both SGI1^Δ*sgiAT*^ and R55 slightly increased compared to that of the SGI1^WT^ context. Using kanamycin selective pressure to maintain IncA/C plasmid R55 in the bacterial population, the loss of SGI1^Δ*sgiAT*^ observed was significantly increased to 90% compared to only about 40% for SGI1^WT^ under the same condition. Nevertheless, under tetracycline selective pressure to maintain SGI1 in the bacterial population, IncA/C plasmid R55 seemed to maintain together with SGI1 to the same extent (around 50% of the bacterial population), whether *sgiAT* is present or not (Fig. 3a), suggesting that the incompatibility between these genetic elements is not dependent on *sgiAT*.

The loss of SGI1 in absence of antibiotic selective pressure was further measured by qPCR on the whole bacterial population (Fig. 3b). Targeting the total SGI1 copy number, the qPCR data confirmed a SGI1 loss of ca. 40% when *sgiAT* is deleted. Moreover, this result was further confirmed by less integrated- and excised-forms of SGI1 and more empty integration *attB* sites in the SGI1^Δ*sgiAT*^ bacterial population compared to the SGI1^WT^-carrying strain (Fig. 3b). Targeting the different forms of SGI1 and the IncA/C plasmid R55, the qPCR data were in agreement with the data described above confirming the role of the *sgiAT* system in the maintenance of SGI1 in the bacterial population.

All together these data suggest that the *sgiAT* system plays an important role in the stable SGI1 maintenance in bacterial populations when an IncA/C plasmid is concomitantly present. *sgiAT* in terms of incompatibility between the two elements seems to act primarily on stability of SGI1 and this stable maintenance may favor as a consequence the loss of the IncA/C plasmid.

Another important observation regarding incompatibility between SGI1 and IncA/C, is that we were unable to stably maintain plasmid R55 in a SGI1 Δ*int* strain. *int* is the first gene of SGI1 and encodes an integrase shown previously to be crucial for SGI1 excision^6^. This situation where SGI1 cannot excise and thus cannot be lost at least through integrase-mediated excision, provides further experimental evidence of incompatibility between these genetic elements.

### The *sgiAT* system is widely distributed

While 412 counterparts of the SgiA antitoxin are found in GenBank, only about half, 187, are detected for the SgiT toxin. Furthermore, all the SgiT homologous proteins found in GenBank are always located next to an homolog of the SgiA antitoxin, thereby confirming a potential coupled action between the two proteins and supporting the hypothesis of the impossibility of harboring a gene encoding the SgiT toxin without the presence of its corresponding antidote. While a few pair encoding genes are located on some plasmids (15%), all the others are located on the chromosome in the vicinity of genomic island-related genes suggesting that these *sgiAT* homologs may participate in their stabilization in the genome. This TA system is not confined to *Proteobacteria*, as initially observed in *Agrobacterium tumefaciens* or *Salmonella enterica*, but as shown in the Supplemental Figure 2 phylogenetic tree it occurs largely among the major phyla of *Eubacteria: Bacteroidetes, Firmicutes, Cyanobacteria, Actinobacteria, Armatimonadetes, Deferribacteres* or *Spirochaetes*.

## Concluding Remarks

In this study the SGI1 orfs S025 and S026 were shown to encode a TA system involved in stable maintenance of this island, and this TA system was thus named *sgiTA*. In the absence of IncA/C plasmid in the SGI1-carrying host cell, the constitutive expression of the SGI1 integrase stably maintains the island integrated into the chromosome^5^,^8^. The IncA/C-encoded transcriptional activator complex AcaCD triggers the SGI1 excision and *in trans* conjugative mobilization^4^,^5^,^6^,^7^,^8^,^9^,^10^. In addition, among the most prevalent Inc groups of plasmid families, only the IncA/C plasmid family has been shown to mediate transfer of SGI1^9^. Thus in the concomitant presence of an IncA/C plasmid, when SGI1 is extrachromosomal and more likely to be lost, *sgiTA* may play in this situation an important role in SGI1 stability within the bacterial population to reduce the formation of SGI1-free cells. Considering the fact that SGI1 requires specifically an IncA/C plasmid for its horizontal transfer, the observation of incompatibility in this study between these two elements may look paradoxical. To the best of our knowledge, this study represents the first description of incompatibility between a plasmid family and an integrative mobilizable element. This incompatibility phenomenon may epidemiologically explain why SGI1 and IncA/C plasmids are not found simultaneously in the same MDR clones. Other genetic factors behind this subtle interplay of conjugative transfer and incompatibility remain to be identified. *sgiAT* counterparts seem to be widely distributed in the bacterial world on other chromosomal islands and plasmids as well, and may thus act as stabilizer systems for these elements in their respective bacterial host. In addition, they possibly ensure stable maintenance of other functional properties to their bacterial host than antimicrobial resistance.

## Materials and Methods

### Bacterial strains, plasmids and media

Strains and plasmids used are listed in Table 1. *S. enterica* serovar Agona (*S.* Agona) strain 959SA97 was used as SGI1-carrying strain^2^. *E. coli* MG1655 derivative strain DJ480 was used in the toxicity-antitoxicity assays^37^. *E. coli* strain TOP10 was used for the PSK assays (Invitrogen SARL, Cergy-Pontoise, France). Plasmid R55 was used as the reference IncA/C plasmid in SGI1 stability and compatibility tests. For classical culture conditions, Brain-Heart-Infusion (BHI) and Luria-Bertani (LB) broth and agar media were used. For toxicity-antitoxicity assays, Ceria 132 synthetic medium (CM) and CM supplemented with 0.1% Casamino Acids (CCM) with or without 1% glucose and with or without 1% arabinose were used according to Glansdorff *al.*^38^. For PSK assays RM liquid medium (Invitrogen) supplemented with 2.5% arabinose, to induce vector expression, was used. The *Salmonella*-*Shigella* (SS) medium with addition of appropriate antibiotics was used for the selection of *S*. Agona 959SA97 – plasmid R55 transconjugant strains. Antibiotics were used in liquid and agar media at the following concentrations: Chloramphenicol (Chl) (30 μg/mL), Kanamycin (Kan) (50 μg/mL), Gentamicin (Gen) (20 μg/mL), Tetracycline (Tet) (30 μg/mL), Ampicillin (Amp) (50 μg/mL) and Spectinomycin (Spc) (100 μg/mL).

### Construction of recombinant plasmids

Plasmid vectors pBAD33 and pKK223-3 were used to directionally clone and express S025 and S026 under the control of their arabinose *P*_*BAD*_ promoter and *P*_*tac*_ promoter, respectively (Table 1)^39^,^40^. Briefly, the S026 orf was amplified by PCR using the 5′-S026-EcoRI and 3′-S026-PstI primers (Fig. 1, Supplementary Table 1). The PCR product was cloned into the pCR2.1 TA cloning vector (Invitrogen). By EcoRI-PstI digestion, the insert of the recombinant plasmid was further subcloned into pKK223-3, resulting in plasmid named pKH02. The S025 orf with its putative RBS was amplified by PCR using the 5′-S025-SacI and 3′-S025-XbaI primers (Fig. 1, Supplementary Table 1). The PCR product was cloned into the pBAD-TOPO cloning vector (Table 1). The SacI-XbaI fragment containing S025 was then subcloned into pBAD33, resulting in plasmid pKH01 (Table 1).

Plasmid vector pMLO59 was used to clone the entire S026-S025 region with its own promoter region predicted by using the Softberry BPROM tool (http://www.softberry.com/berry.phtml?topic=bprom&group=programs&subgroup=gfindb) (the putative promoter region is indicated in Fig. 1). This region was amplified by PCR using the FwOpS026 and RvOpS025 primers (Fig. 1, Supplementary Table 1), and cloned into the pCR2.1 TA cloning vector (Invitrogen). The EcoRI fragment of this recombinant plasmid, containing S026-S025, was then subcloned into the pMLO59 plasmid vector^31^, resulting in recombinant plasmid pKH03 (Table 1). The S026-S025 region was also amplified by PCR without its putative promoter region, using the FwOPS026S025-KpnI and RvOpS026S025-SalI primers (Supplementary Table 1). The PCR product was cloned into the pBAD-TOPO cloning vector (Invitrogen). The KpnI-SalI fragment of the resulting recombinant plasmid, containing S026-S025, was then subcloned into plasmid pBAD33. The ClaI-SalI insert of the resulting recombinant plasmid, containing the AraC gene and the *P*_*BAD*_ promoter followed by S026-S025 (the pBAD33 *AraC* gene encodes the transcriptional activator of *P*_*BAD*_ in the presence of arabinose), was then subcloned into pMLO59 and resulted in recombinant plasmid pKH04 (Table 1). All cloned fragments were verified by DNA sequencing (Cogenics, Grenoble, France).

### Deletion of the S026-S025 region

Deletion of the entire SGI1 S026-S025 region was performed in *S.* Agona 959SA97 by use of the one-step chromosomal gene inactivation technique of Datsenko & Wanner^41^. Briefly, the kanamycin resistance gene kan flanked by FRT sites was amplified by PCR using the template plasmid pKD4 and hybrid primers (Table 1). These primers, Fw∆S026-S025 and Fw∆S026-S025 (Supplementary Table 1), were synthesized with 20 nucleotides of priming sites of pKD4 and with 50 nucleotides from each side of the S026-S025 operon. The 1.6 kb PCR product was purified and electroporated into *S.* Agona 959SA97 in which the λ Red recombinase expression plasmid pKD46-Gm was introduced^42^. Homologous recombination between the PCR product and the genomic DNA resulted in the deletion of a 3.8 kb region, comprising S026-S025, and in its replacement by the kan resistance cassette (Fig. 1). This mutant strain was named 959SA97ΔS026-S025::kan. Different PCRs were performed to confirm that the SGI1 mutant strains had the deletion correctly located, using the set of primers k2, kt, FwS026-S025, and RvS026-S025 (Fig. 1, Table 1)^41^. To eliminate the kan cassette, the Kan-resistant mutant strain 959SA97ΔS026-S025::kan was transformed with the FLP recombinase expression plasmid pCP20-Gm (oriR temperature sensitive^41^), and gentamicin-resistant transformants were selected at 30 °C. After culture at 37 °C and/or 43 °C, a large majority of transformants had lost the FRT flanked kan gene and also the FLP helper plasmid pCP20-Gm and thus became susceptible to both kanamycin and gentamicin. The resulting mutant strain named 959SA97ΔS026S025::FRT was confirmed by PCR using primers flanking the deleted region and nucleotide sequencing revealed that it harboured a 85 nucleotides scar in place of S026-S025 as expected^41^. The same methodology as described above was used to construct S025 and S026 deletion mutant strains, i.e. 959SA97ΔS025::FRT and 959SA97ΔS026::kan, respectively.

### Bacterial conjugation experiments

Bacterial conjugation, performed as described previously^9^, was used to introduce the IncA/C R55 plasmid into *S*. Agona 959SA97 or 959SA97ΔS026-S025::FRT. Briefly, end-log exponential phase liquid cultures in BHI medium of an *E. coli* donor strain containing plasmid pR55 and of *S.* Agona strain 959SA97 or 959SA97ΔS026-S025::FRT were mixed together at a 1:4 ratio (1 mL of donor strain for 4 mL of recipient strain). After overnight incubation without shaking at 37 °C, the mating was plated on SS agar plates supplemented with appropriate antibiotics for SGI1 and R55 selection (i.e. Kan at 50 μg/mL or Tet at 10 μg/mL). All transconjugant strains were confirmed by PCR and stored at −80 °C.

### Toxicity-antitoxicity assays

To assess if S026-S025 encodes a TA system, S025 and S026 were cloned separately or together, depending on the experiment, in plasmid vectors allowing expression under the control of inducible promoters. The following experiments were performed according to previous studies on TA systems^30^,^31^,^32^.

#### (i) Transformation efficiency

*E. coli* DJ480 strains carrying the pKK223-3 vector^43^ or its derivative expressing S026 (putative antitoxin) (plasmid pKH01) were transformed with 1 ng of pBAD33 vector or its pKH02 (putative S025 toxin) derivative. After 3 hours of incubation at 37 °C in SOC medium, transformation mixtures were plated on LB with appropriate antibiotics (Amp at 50 μg/mL and Chl at 30 μg/mL) with or without arabinose (0%, 0.2% or 1% of arabinose). Plates were incubated overnight at 37 °C. The efficiency of transformation was calculated as the number of transformant CFUs obtained on arabinose plates for 1.10^7^ plasmid copies.

Overnight cultures of *E. coli* DJ480 strains carrying pKH02 (encoding the putative toxin S025) and the pKK223-3 vector or its derivative expressing the putative antitoxin S026 (pKH01) were inoculated at 0.01 final OD_600nm_ in 100 mL of CCM medium supplemented with 1% glycerol and the appropriate antibiotics (Cm 30 μg/mL for pBAD33 and pKH02; Amp 50 μg/mL for pKK223-3 and pKH01). These cultures were grown at 37 °C to an OD_600nm_ of 0.15 and then divided in 3 subcultures of 25 mL with or without addition of arabinose for induction (0%, 0.2% or 1%). Sampling was performed at 0, 10, 20, 30, 40, 60, and 120 minutes, and serial dilutions were plated on CCM medium with appropriate antibiotics and glucose 0.4% to stop the S025 expression. In addition, 10 μL drops of 10-fold serial dilutions of these *E. coli* DJ480 strains were deposited on LB agar medium with or without arabinose at a final concentration of 1%. After overnight incubation at 37 °C plates were photographed.

#### (iii) PSK assay

To assess if S026-S025 induces a PSK effect, the putative operon S026-S025 with its own putative promoter or under control of the *P*_*BAD*_ promoter was cloned in plasmid pMLO59 as described above, resulting in recombinant plasmids pKH03 and pKH04, respectively. Plasmid pMLO59 has a thermosensitive origin of replication^31^, and thus it replicates well at 30 °C but not at 42 °C. The *E. coli* TOP10 strains containing the empty pMLO59 vector or derivate plasmids pKH03 or pKH04 were grown overnight at 30 °C in RM liquid medium containing Spc (100 μg/ml) and arabinose at 1% final concentration for pKH04 (to induce TA expression). Overnight cultures were diluted 10,000-fold in RM prewarmed at 42 °C with 2.5% arabinose for pKH04. Samples were plated on LB agar plates with or without Spc (50 μg/ml) at different times (0 min., 80 min., 170 min., 280 min., 360 min., 485 min., 540 min. for pKH04 and 0 min., 70 min., 110 min., 180 min., 260 min., 305 min. for pKH03). Plates were incubated overnight at 30 °C, and CFUs were enumerated.

### SGI1 stability and compatibility tests

The maintenance of SGI1 and compatibility with IncA/C plasmid were assessed in *S*. Agona 959SA97 derivative strains carrying or not SGI1^WT^, SGI1^∆S026S025^, and the IncA/C plasmid R55. These strains were conserved at −80 °C after culture with antibiotic selective pressure for both elements if needed. Briefly, strains conserved at −80 °C were directly plated on LB agar plates and incubated at 37 °C for 16 h. Colonies obtained were tested by PCR for the presence of SGI1 and plasmid R55 using primers in Supplementary Table 1. Then 10 positive colonies for both elements were pooled to constitute one sample. The bacterial population of each sample was then tested for susceptibility to Kan or Tet to quantify the loss of each genetic element, as follows. Samples diluted at 1/100 were plated on LB agar and incubated for 16 h at 37 °C. CFUs obtained were then replicated on Kan containing LB plates, on Tet containing LB plates, and finally on LB plates without antibiotics. After overnight culture at 37 °C, each colony obtained was further checked for growth. Absence of growth on Kan and/or Tet LB plates indicated the loss of either R55 and/or SGI1, respectively. For each sample DNA was also extracted to perform qPCR, using primers listed in Supplementary Table 1, to quantify the loss of each genetic element. qPCR was performed on bio-rad DyadDisciple Chromo4 Real time PCR detector with the Bio-rad IQ Sybr Green supermix (BioRad, Marne La Coquette, France).

### Distribution of *sgiAT*

Protein sequences of S025 and S026, with GenBank accession numbers ACS32052 and ACS32053 respectively, where used as tblastn queries to search for similar sequences in GenBank. Homologous protein sequences where retrieved with genome id, coordinates and taxonomic unit. Homologous proteins from a same genome and distant of less than 1 kb were conserved for further analysis. To classify the genomic region encoding couples of SgiAT homologous proteins, sequence header was analyzed for the presence of the word “plasmid” and 30 kb surrounding regions were analyzed for the presence of genomic island related genes. Sequences were aligned with clustalo and maximum-likelihood phylogenetic trees were constructed by phyML under a LG model with optimal tree structure from the best of NNI and SPR. In accordance with a reproducible research approach, data, python scripts and figures are available at https://github.com/MathGon/SGI_TA.

## Additional Information

**How to cite this article**: Huguet, K. T. *et al*. A toxin antitoxin system promotes the maintenance of the IncA/C-mobilizable *Salmonella* Genomic Island 1. *Sci. Rep.* **6**, 32285; doi: 10.1038/srep32285 (2016).

## Supplementary Material

Supplementary Information

## Figures and Tables

**Table 1:** Strains and plasmids used in this study.

Strain or plasmid	Relevant genotype, antimicrobial resistance profile, or plasmid properties	Used for	Reference or source
*Salmonella enterica*			
Agona 959SA97	Wild-type, SGI1; AmpChlFfcStrSptSulTet	WT strain	6
Agona 959SA97^∆S026-S025::FRT^	SGI1∆^S026-S025::FRT^; AmpChlFfcStrSptSulTet	SGI1 stability test	This work
Agona 959SA97∆^S025::FRT^	SGI1∆^S025::FRT^; AmpChlFfcStrSptSulTet	SGI1 stability test	This work
Agona 959SA97∆^S026::Kan^	SGI1∆^S026::Kan^; AmpChlFfcStrSptSulTetKan	S025 toxicity test	This work
Agona 959SA97∆^SGI1^	Loss of SGI1 through excision; Str	IncA/C R55 stability test	This work
*E. coli*			
DJ480	MG1655 Δ*lac*X74 Δ*ara, mal*P::*lac*IQ	Cloning and TA test	31,32
TOP10	F- *mcr*A Δ(*mrr-hsd*RMS-*mcr*BC) f80*lac*ZΔM15 Δ*lac*X74 *rec*A1 *ara*D139 Δ(*ara-leu*)7697 *gal*U *gal*K *rps*L(StrR) *end*A1 *nup*G	Cloning and TA test	Invitrogen®
Plasmids			
pCR2.1	TOPO cloning vector	Cloning	Invitrogen®
pBAD-TOPO	TOPO cloning vector	Cloning	Invitrogen®
IncA/C R55	tra+; AmpChlFfcGenKanSul	SGI1 stability test	6,9
pBAD33	p15A ori. arabinose *P*_*BAD*_ promoter, Chl	TA test	31,32
pKK223-3	ColE1 ori, *P*_*tac*_ promoter, Amp	TA test	31,32
pMLO59	pGB2 thermosensitive derivative, Spt	PSK assay	31,32
pKH01	S026 cloned in pKK223-3 under the control of the *P*_*tac*_ promoter	TA test	This work
pKH02	S025 cloned in pBAD33 under the control of the *P*_*BAD*_ promoter, Chl	TA test	This work
pKH03	S026-S025 cloned in pMLO59 under the control of their own putative promoter	PSK assay	This work
pKH04	S026-S025 cloned in pMLO59 under the control of the *P*_*BAD*_ promoter	PSK assay	This work

**Table 2:** Independent maintenance of SGI1, mutant derivatives and IncA/C plasmid R55 in *S.* Agona strain 959SA97.

	Bacterial generation no.	SGI1^WT^	SGI1^Δ*int*^	SGI1^Δ*sgiAT*^	IncA/C R55
		CFU/ml			
phenotypic detection§	9	3.05 10^10^ (1.60 10^10^)£	3.90 10^10^ (1.95 10^10^)	7.30 10^10^ (4.15 10^10^)	ND
	234	2.39 10^10^ (2.09 10^10^)	1.81 10^10^ (1.31 10^10^)	1.98 10^10^ (2.29 10^10^)	ND
	351	1.24 10^10^ (1.78 10^10^)	1.60 10^10^ (1.00 10^10^)	1.98 10^10^ (9.25 10^9^)	ND
Molecular detection¥	18	100/100	ND	100/100	97/100

ND, not determined.

^§^The presence of SGI1 or mutant derivatives was monitored by plating serial dilutions on LB agar with or without kanamycin (resistance conferred by SGI1) during 351 bacterial generations as performed according to Kiss *et al*.^7^.

^¥^Bacteria carrying SGI1 or mutant derivatives and total bacteria were scored as the number of CFU/ml and are indicated in uppercase and lowercase (in parentheses) numbers, respectively.

^£^After 18 generations, dilutions were plated on LB agar. After O/N cultures at 37 °C, 100 CFUs were tested by PCR for the presence of SGI1, mutant derivatives or the IncA/C plasmid R55. The maintenance of R55 was assessed in *S.* Agona strain 959SA97^ΔSGI1^.
