# Supplementary material for: A toxin antitoxin system promotes the maintenance of the IncA/C-mobilizable Salmonella Genomic Island 1
Source: Sci Rep. 2016 Aug 31;6:32285. doi: 10.1038/srep32285 (PMC5006074; doi:10.1038/srep32285)
Supplement: Supplementary Information [file srep32285-s1.pdf]

A toxin antitoxin system promotes the maintenance of the IncA/C-mobilizable *Salmonella* Genomic Island 1

Kevin T. Huguet[1,2], Mathieu Gonnet[1,2], Benoît Doublet[1,2], and Axel Cloeckaert[1,2]

[1] INRA, UMR1282, Infectiologie et Santé Publique, F-37380 Nouzilly, France

[2] Université François Rabelais de Tours, UMR1282 Infectiologie et Santé Publique, F-37000 Tours, France

Corresponding author: Axel Cloeckaert (email: Axel.Cloeckaert@tours.inra.fr)

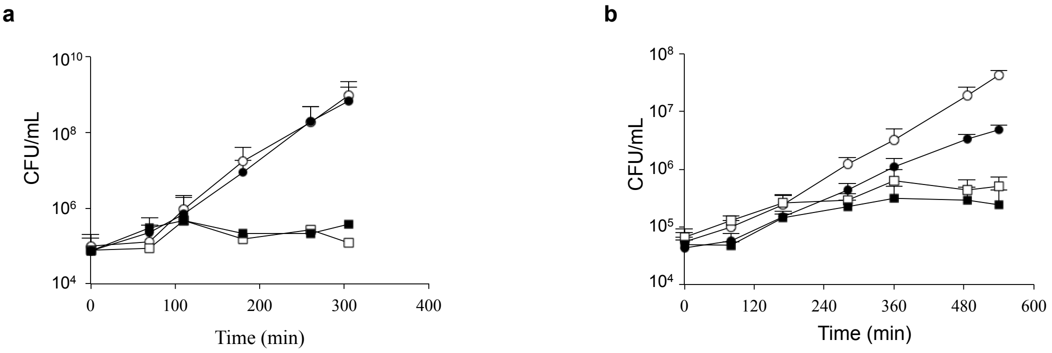

Supplementary Figure 1. Post segregational killing assays in *E. coli* TOP10.
(**a**) Curves with empty symbols represent growth of *E. coli* carrying the pMLO59 replication-thermosensitive vector (spectinomycin resistance) cultured in RM medium at 42 °C. Curves with filled symbols represents growth of *E. coli* carrying pKH03 (pMLO59 containing the putative operon S026-S025 under the control of its own putative promoter) cultured in RM medium at 42 °C. Viability (CFU/ml) was monitored for 300 min. by plating serial dilutions on LB agar with (squares) or without (circles) spectinomycin. (**b**) Curves with empty symbols represent growth of *E. coli* carrying pMLO59 empty vector cultured in RM medium at 42 °C supplemented with 2.5 % arabinose. Curves with filled symbols represents growth of *E. coli* carrying pKH04 (pMLO59 containing S026-S025 under the control of the P^{ARA} promoter) cultured in RM medium at 42 °C supplemented with 2.5 % arabinose. Viability (CFU/ml) was monitored for 540 min. by plating serial dilutions on LB agar with (squares) or without (circles) spectinomycin and incubated at 30°C. Values correspond to the mean of three independent experiments.

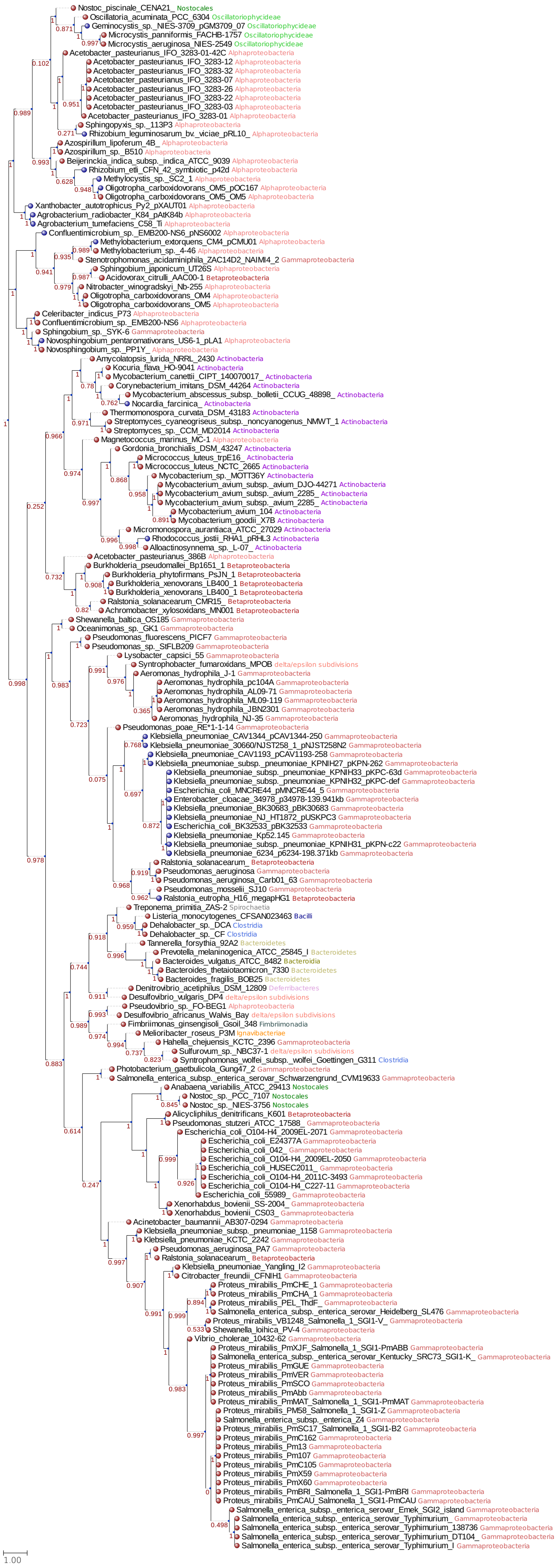

Nostoc_piscinale_CENA21_ Nostocales
Oscillatoria_acuminata_PCC_6304 Oscillatoriophycideae
Geminocystis_sp._NIES-3709_pGM3709_07 Oscillatoriophycideae
Microcystis_panniformis_FACHB-1757 Oscillatoriophycideae
Microcystis_aeruginosa_NIES-2549 Oscillatoriophycideae
Acetobacter_pasteurianus_IFO_3283-01-42C Alphaproteobacteria
Acetobacter_pasteurianus_IFO_3283-12 Alphaproteobacteria
Acetobacter_pasteurianus_IFO_3283-32 Alphaproteobacteria
Acetobacter_pasteurianus_IFO_3283-07 Alphaproteobacteria
Acetobacter_pasteurianus_IFO_3283-26 Alphaproteobacteria
Acetobacter_pasteurianus_IFO_3283-22 Alphaproteobacteria
Acetobacter_pasteurianus_IFO_3283-03 Alphaproteobacteria
Acetobacter_pasteurianus_IFO_3283-01 Alphaproteobacteria
Sphingopyxis_sp._113P3 Alphaproteobacteria
Rhizobium_leguminosarum_bv._viciae_pRL10_ Alphaproteobacteria
Azospirillum_lipoferum_4B_ Alphaproteobacteria
Azospirillum_sp._B510 Alphaproteobacteria
Beijerinckia_indica_subsp._indica_ATCC_9039 Alphaproteobacteria
Rhizobium_etli_CFN_42_symbiotic_p42d Alphaproteobacteria
Methylocystis_sp._SC2_1 Alphaproteobacteria
Oligotropha_carboxidovorans_OM5_pOC167 Alphaproteobacteria
Oligotropha_carboxidovorans_OM5_OM5 Alphaproteobacteria
Xanthobacter_autotrophicus_Py2_pXAUT01 Alphaproteobacteria
Agrobacterium_radiobacter_K84_pAtK84b Alphaproteobacteria
Agrobacterium_tumefaciens_C58_Ti Alphaproteobacteria
Confluentimicrobium_sp._EMB200-NS6_pNS6002 Alphaproteobacteria
Methylobacterium_extorquens_CM4_pCMU01 Alphaproteobacteria
Methylobacterium_sp._4-46 Alphaproteobacteria
Stenotrophomonas_acidaminiphila_ZAC14D2_NAIMI4_2 Gammaproteobacteria
Sphingobium_japonicum_UT26S Alphaproteobacteria
Acidovorax_citrulli_AAC00-1 Betaproteobacteria
Nitrobacter_winogradskyi_Nb-255 Alphaproteobacteria
Oligotropha_carboxidovorans_OM4 Alphaproteobacteria
Oligotropha_carboxidovorans_OM5 Alphaproteobacteria
Celeribacter_indicus_P73 Alphaproteobacteria
Confluentimicrobium_sp._EMB200-NS6 Alphaproteobacteria
Sphingobium_sp._SYK-6 Gammaproteobacteria
Novosphingobium_pentaromativorans_US6-1_pLA1 Alphaproteobacteria
Novosphingobium_sp._PP1Y_ Alphaproteobacteria
Amycolatopsis_lurida_NRRL_2430 Actinobacteria
Kocuria_flava_HO-9041 Actinobacteria
Mycobacterium_canettii_CIPT_140070017_ Actinobacteria
Corynebacterium_imitans_DSM_44264 Actinobacteria
Mycobacterium_abscessus_subsp._bolletii_CCUG_48898_ Actinobacteria
Nocardia_farcinica_ Actinobacteria
Thermomonospora_curvata_DSM_43183 Actinobacteria
Streptomyces_cyaneogriseus_subsp._noncyanogenus_NMWT_1 Actinobacteria
Streptomyces_sp._CCM_MD2014 Actinobacteria
Magnetococcus_marinus_MC-1 Alphaproteobacteria
Gordonia_bronchialis_DSM_43247 Actinobacteria
Micrococcus_luteus_trpE16_ Actinobacteria
Micrococcus_luteus_NCTC_2665 Actinobacteria
Mycobacterium_sp._MOTT36Y Actinobacteria
Mycobacterium_avium_subsp._avium_DJO-44271 Actinobacteria
Mycobacterium_avium_subsp._avium_2285_ Actinobacteria
Mycobacterium_avium_subsp._avium_2285_ Actinobacteria
Mycobacterium_avium_104 Actinobacteria
Mycobacterium_goodii_X7B Actinobacteria
Micromonospora_aurantiaca_ATCC_27029 Actinobacteria
Rhodococcus_jostii_RHA1_pRHL3 Actinobacteria
Alloactinosynnema_sp._L-07_ Actinobacteria
Acetobacter_pasteurianus_386B Alphaproteobacteria
Burkholderia_pseudomallei_Bp1651_1 Betaproteobacteria
Burkholderia_phytofirmans_PsJN_1 Betaproteobacteria
Burkholderia_xenovorans_LB400_1 Betaproteobacteria
Burkholderia_xenovorans_LB400_1 Betaproteobacteria
Ralstonia_solanacearum_CMR15_ Betaproteobacteria
Achromobacter_xylosoxidans_MN001 Betaproteobacteria
Shewanella_baltica_OS185 Gammaproteobacteria
Oceanimonas_sp._GK1 Gammaproteobacteria
Pseudomonas_fluorescens_PICF7 Gammaproteobacteria
Pseudomonas_sp._StFLB209 Gammaproteobacteria
Lysobacter_capsici_55 Gammaproteobacteria
Syntrophobacter_fumaroxidans_MPOB delta/epsilon subdivisions
Aeromonas_hydrophila_J-1 Gammaproteobacteria
Aeromonas_hydrophila_pc104A Gammaproteobacteria
Aeromonas_hydrophila_AL09-71 Gammaproteobacteria
Aeromonas_hydrophila_ML09-119 Gammaproteobacteria
Aeromonas_hydrophila_JBN2301 Gammaproteobacteria
Aeromonas_hydrophila_NJ-35 Gammaproteobacteria
Pseudomonas_poae_RE*1-1-14 Gammaproteobacteria
Klebsiella_pneumoniae_CAV1344_pCAV1344-250 Gammaproteobacteria
Klebsiella_pneumoniae_30660/NJST258_1_pNJST258N2 Gammaproteobacteria
Klebsiella_pneumoniae_CAV1193_pCAV1193-258 Gammaproteobacteria
Klebsiella_pneumoniae_subsp._pneumoniae_KPNIH27_pKPN-262 Gammaproteobacteria
Klebsiella_pneumoniae_subsp._pneumoniae_KPNIH33_pKPC-63d Gammaproteobacteria
Klebsiella_pneumoniae_subsp._pneumoniae_KPNIH32_pKPC-def Gammaproteobacteria
Escherichia_coli_MNCRE44_pMNCRE44_5 Gammaproteobacteria
Enterobacter_cloacae_34978_p34978-139.941kb Gammaproteobacteria
Klebsiella_pneumoniae_BK30683_pBK30683 Gammaproteobacteria
Klebsiella_pneumoniae_NJ_HT1872_pUSKPC3 Gammaproteobacteria
Escherichia_coli_BK32533_pBK32533 Gammaproteobacteria
Klebsiella_pneumoniae_Kp52.145 Gammaproteobacteria
Klebsiella_pneumoniae_subsp._pneumoniae_KPNIH31_pKPN-c22 Gammaproteobacteria
Klebsiella_pneumoniae_6234_p6234-198.371kb Gammaproteobacteria
Ralstonia_solanacearum_ Betaproteobacteria
Pseudomonas_aeruginosa Gammaproteobacteria
Pseudomonas_aeruginosa_Carb01_63 Gammaproteobacteria
Pseudomonas_mosselii_SJ10 Gammaproteobacteria
Ralstonia_eutropha_H16_megapHG1 Betaproteobacteria
Treponema_primitia_ZAS-2 Spirochaetia
Listeria_monocytogenes_CFSAN023463 Bacilli
Dehalobacter_sp._DCA Clostridia
Dehalobacter_sp._CF Clostridia
Tannerella_forsythia_92A2 Bacteroidetes
Prevotella_melaninogenica_ATCC_25845_I Bacteroidetes
Bacteroides_vulgatus_ATCC_8482 Bacteroidia
Bacteroides_thetaiotaomicron_7330 Bacteroidetes
Bacteroides_fragilis_BOB25 Bacteroidetes
Denitrovibrio_acetiphilus_DSM_12809 Deferribacteres
Desulfovibrio_vulgaris_DP4 delta/epsilon subdivisions
Pseudovibrio_sp._FO-BEG1 Alphaproteobacteria
Desulfovibrio_africanus_Walvis_Bay delta/epsilon subdivisions
Fimbriimonas_ginsengisoli_Gsoil_348 Fimbriimonadia
Melioribacter_roseus_P3M Ignavibacteriae
Hahella_chejuensis_KCTC_2396 Gammaproteobacteria
Sulfurovum_sp._NBC37-1 delta/epsilon subdivisions
Syntrophomonas_wolfei_subsp._wolfei_Goettingen_G311 Clostridia
Photobacterium_gaetbulicola_Gung47_2 Gammaproteobacteria
Salmonella_enterica_subsp._enterica_serovar_Schwarzengrund_CVM19633 Gammaproteobacteria
Anabaena_variabilis_ATCC_29413 Nostocales
Nostoc_sp._PCC_7107 Nostocales
Nostoc_sp._NIES-3756 Nostocales
Alicycliphilus_denitrificans_K601 Betaproteobacteria
Pseudomonas_stutzeri_ATCC_17588_ Gammaproteobacteria
Escherichia_coli_O104-H4_2009EL-2071 Gammaproteobacteria
Escherichia_coli_E24377A Gammaproteobacteria
Escherichia_coli_042_ Gammaproteobacteria
Escherichia_coli_O104-H4_2009EL-2050 Gammaproteobacteria
Escherichia_coli_HUSEC2011_ Gammaproteobacteria
Escherichia_coli_O104-H4_2011C-3493 Gammaproteobacteria
Escherichia_coli_O104-H4_C227-11 Gammaproteobacteria
Escherichia_coli_55989_ Gammaproteobacteria
Xenorhabdus_bovienii_SS-2004_ Gammaproteobacteria
Xenorhabdus_bovienii_CS03_ Gammaproteobacteria
Acinetobacter_baumannii_AB307-0294 Gammaproteobacteria
Klebsiella_pneumoniae_subsp._pneumoniae_1158 Gammaproteobacteria
Klebsiella_pneumoniae_KCTC_2242 Gammaproteobacteria
Pseudomonas_aeruginosa_PA7 Gammaproteobacteria
Ralstonia_solanacearum_ Betaproteobacteria
Klebsiella_pneumoniae_Yangling_I2 Gammaproteobacteria
Citrobacter_freundii_CFNIH1 Gammaproteobacteria
Proteus_mirabilis_PmCHE_1 Gammaproteobacteria
Proteus_mirabilis_PmCHA_1 Gammaproteobacteria
Proteus_mirabilis_PEL_ThdF_ Gammaproteobacteria
Salmonella_enterica_subsp._enterica_serovar_Heidelberg_SL476 Gammaproteobacteria
Proteus_mirabilis_VB1248_Salmonella_1_SGI1-V_ Gammaproteobacteria
Shewanella_loihica_PV-4 Gammaproteobacteria
Vibrio_cholerae_10432-62 Gammaproteobacteria
Proteus_mirabilis_PmXJF_Salmonella_1_SGI1-PmABB Gammaproteobacteria
Salmonella_enterica_subsp._enterica_serovar_Kentucky_SRC73_SGI1-K_ Gammaproteobacteria
Proteus_mirabilis_PmGUE Gammaproteobacteria
Proteus_mirabilis_PmVER Gammaproteobacteria
Proteus_mirabilis_PmSCO Gammaproteobacteria
Proteus_mirabilis_PmAbb Gammaproteobacteria
Proteus_mirabilis_PmMAT_Salmonella_1_SGI1-PmMAT Gammaproteobacteria
Proteus_mirabilis_PM58_Salmonella_1_SGI1-Z Gammaproteobacteria
Salmonella_enterica_subsp._enterica_Z4 Gammaproteobacteria
Proteus_mirabilis_PmSC17_Salmonella_1_SGI1-B2 Gammaproteobacteria
Proteus_mirabilis_PmC162 Gammaproteobacteria
Proteus_mirabilis_Pm13 Gammaproteobacteria
Proteus_mirabilis_Pm107 Gammaproteobacteria
Proteus_mirabilis_PmC105 Gammaproteobacteria
Proteus_mirabilis_PmX59 Gammaproteobacteria
Proteus_mirabilis_PmX60 Gammaproteobacteria
Proteus_mirabilis_PmBRI_Salmonella_1_SGI1-PmBRI Gammaproteobacteria
Proteus_mirabilis_PmCAU_Salmonella_1_SGI1-PmCAU Gammaproteobacteria
Salmonella_enterica_subsp._enterica_serovar_Emek_SGI2_island Gammaproteobacteria
Salmonella_enterica_subsp._enterica_serovar_Typhimurium_ Gammaproteobacteria
Salmonella_enterica_subsp._enterica_serovar_Typhimurium_138736 Gammaproteobacteria
Salmonella_enterica_subsp._enterica_serovar_Typhimurium_DT104_ Gammaproteobacteria
Salmonella_enterica_subsp._enterica_serovar_Typhimurium_I Gammaproteobacteria
1.00

Supplementary Figure 2. Maximum-Likelihood phylogenetic tree of S025-S026 homologous protein sequences concatenation. Leaves are labeled with strain names, phyla and a pellet determining the type of replicon: blue for a plasmid and red for a genomic island. Internal nodes are labeled with the bootstrap value.

Supplementary Table 1. Sequences of the primers used in this study.

Primer	Séquence (5'-3')[§]	Position in SGI1 (GenBank accession no. AF261825)
Cloning		
Fw5'-S025-SacI	*GAGCTC*AGGAGGATTATATGGTTTCTAAAAG	24879-24897
Rv3'-S025-XbaI	AGTCT*TCTAGA*TCAAGTTACAATTTCTAT	22349-22366
Fw5'-S026-EcoRI	TTGT*GAATTC*AATGGCTACTGCGGAACA	25886-25903
Rv3'-S026-PstI	AGTCT*CTGCAG*TTATTTGTTATTACCTGACTGC	24913-24934
FwS026-S025	GGTTCTATTCCTGTATCTGA	26066-26085
RvS026-S025	AGTGTCAGTTCTATTGGTTC	22204-22223
FwS026-S025-KpnI	TCGA*GGTACC*TGGTAAAGTTGCCCCGTCTT	25917-25936
RvS026-S025-SalI	GTCA*GTCGAC*AGTGTCAGTTCTATTGGTTC	22204-22223
Control		
U7-L12	ACACCTTGAGCAGGGCAAAG	1-20
LJ-R1	AGTTCTAAAGGTTCGTAGTCG	480-500
Fw-A/C	GAGAACCAAAGACAAAGACCTGGA	NA
Rv-A/C	ACGACAAACCTGAATTGCCTCCTT	NA
FwpBAD	ATGCCATAGCATTTTTATCC	NA
RvpBAD	GATTTAATCTGTATCAGG	NA
FwM13	GTAAAACGACGGCCAGT	NA
RvM13	AACAGCTATGACCATG	NA
Construction of deletion mutants		
FwΔS026-S025	TCAAGTTACAATTTCTATTGCGACCTGATTTTCTATT GCAACTTCAATCT*GTGTAGGCTGGAGCTGCTTC*	22349-22398
FwΔS026	CACATCTAAATGCTTCCTTTTAGAAACCATATAATTC CCTTATATACTTT*GTGTAGGCTGGAGCTGCTTC*	24863-24912
RvΔS026-S025	CTTTATTGTATAAAATTGGTAAAGTTGCCCCGTCTTA TGGAGATTGAATA*CATATGAATATCCTCCTTAG*	25903-25952

RvΔS025	TTCTAGAAGGCAGTCAGGTAATAACAAATAAAAAGT ATATAAGGAATTAT*CATATGAATATCCTCCTTAG*	24893-24943
qPCR primers		
Fwq71276.1[¥]	ATGCCACCCTTTACACCCTG	NA
Rvq71277.1[¥]	CTGTTGAGTCTGAGGTTGCG	NA
FwqattB[£]	TACCTCCGACGACCTACTGG	NA
RvqattB[£]	ATCCGGCTTACGGGTTTTG	NA
FwqattP[+]	CGGATGAAGATCAGGGAGAA	42441-42460
RvqattP[+]	GGTTGTTTTGATATGTTTTGAAAGG	196-220
FwqS024	TGACTGAAATGGGGAGCAAC	21964-21983
RvqS024	CCGAAGAAAGGCAGTTATGG	22038-22057
FwqLeft-J	TGGGACGGATTTTCTCCAG	126-144
RvqLeft-J	CGGAGCAAAATCGTGAGAAG	275-294
FwqA/C	TACACGCCATCGCTTATCAG	NA
RvqA/C	CTGCTTACGCTTGTTGGACG	NA

[§] Italic letters represent artificially added restriction sites or priming sites of pKD4.

[¥] To determine chromosomal DNA copy number (GenBank accession no. NC_011149.1)

[£] To determine the SGI1-free attB sites copy number

[+] To determine the copy number of excised SGI1

NA, not applicable
